# In vitro evaluation of antimicrobial photodynamic therapy with photosensitizers and calcium hydroxide on bond strength, chemical composition, and sealing of glass-fiber posts to root dentin

**DOI:** 10.1007/s10103-025-04302-4

**Published:** 2025-01-28

**Authors:** Thalya Fernanda Horsth Maltarollo, Paulo Henrique dos Santos, Henrique Augusto Banci, Mariana de Oliveira Bachega, Beatriz Melare de Oliveira, Marco Hungaro Antonio Duarte, Índia Olinta de Azevedo Queiroz, Rodrigo Rodrigues Amaral, Luciano Angelo Tavares Cintra, Henrico Badaoui Strazzi-Sahyon, Gustavo Sivieri-Araujo

**Affiliations:** 1 Department of Preventive and Restorative Dentistry, Discipline of Endodontics, Araçatuba School of Dentistry, São Paulo State University - UNESP, Araçatuba, SP Brazil; 2 Restorative Dentistry Area, Faculty of Dentistry, University of Toronto, Toronto, ON Canada; 3 Department of Restorative Dentistry, Endodontics and Dental Materials, Bauru School of Dentistry, São Paulo University - USP, SP Bauru, Brazil; 4 Department of Endodontics, Federal University of Santa Catarina, Florianópolis, SC Brazil; 5 College of Medicine and Dentistry, James Cook University, 1/14-88 McGregor Rd, Building D1, 2nd Floor, Smithfield, Cairns, 4878 QLD Australia; 6 Department of Dental Materials and Prosthodontics, Araçatuba School of Dentistry, São Paulo State University – UNESP, SP Araçatuba, Brazil; 7 Department of Prosthodontics and Periodontology, Bauru School of Dentistry, University of São Paulo - USP, SP Bauru, Brazil; 8 Division of Biomaterial and Biomedical Sciences, Department of Oral Rehabilitation and Biosciences, Oregon Health & Science University, Portland, OR USA

**Keywords:** Bond strength, Dentin, Calcium hydroxide, Photochemotherapy, Photosensitizing agents, Resin cements

## Abstract

Investigate the impact of antimicrobial photodynamic therapy (aPDT) using different photosensitizers (PSs) such as indocyanine green (IG), curcumin (CC), and methylene blue (MB), with or without intracanal application of calcium hydroxide (CH), on the push-out bond strength of glass-fiber posts (GFPs) to intraradicular dentin, the chemical composition of the root substrate, and the sealing of the adhesive interface across different thirds of intraradicular dentin. A total of 112 bovine teeth underwent biomechanical preparation and were divided into eight experimental groups (*n* = 14 each): Negative control with deionized water; positive control with deionized water + CH; IG group with indocyanine green and infrared laser; IG + CH group; CC group with curcumin and blue LED; CC + CH group; MB group with methylene blue and red laser; and MB + CH group. The push-out bond strength was measured using a universal testing machine (*n* = 8), and scanning electron microscopy characterized the fracture patterns. Energy dispersive spectroscopy (*n* = 3) analyzed the chemical composition of the dentin substrate, while fluorescence confocal microscopy (*n* = 3) assessed the adhesive interface sealing between the resin cement and root dentin. Data were analyzed using two-way repeated measures ANOVA and the Tukey test for push-out bond strength and chemical composition comparison, with the Kruskal-Wallis and Dunn’s tests (α = 0.05) for adhesive interface sealing. Significant bond strength differences were noted across root thirds and experimental groups (*P* < .05), with the IG + CH group showing the highest cervical bond strength and the IG group the lowest. Apical bond strength was highest in the CC group but lower in the NC and PC groups. Mixed failures predominated, except in the MB + CH group, where adhesive failures prevailed. Elemental composition varied among groups treated with different PSs and CH (*P* < .05), but interface quality, tag formation, and penetration depth showed no significant differences (*P* > .05). Laser-activated 500 mg/L CC combined with CH emerged as a clinically relevant option for root canal decontamination before GFPs luting. aPDT with different PSs and root canal depth influenced the push-out bond strength of GFPs and the chemical composition of root dentin. Curcumin-mediated aPDT at 500 mg/L proved effective, enhancing bond strength and sealing while maintaining consistent dentin composition across depths.

## Introduction

 Despite technological advancements in Endodontics, microbial persistence within the root canal system (RCS) remains challenging due to the complex internal anatomy and structural resilience of the root canal [1, 2]. Microbial contamination can extend into dentinal tubules, making mechanical and chemical decontamination methods inadequate, especially in complex anatomies like accessory canals and isthmuses. Factors such as irrigation solutions, intracanal medications, and instrumentation type (manual or mechanized) affect endodontic treatment success [3] and the durability of intraradicular support for prosthetic restorations [4, 5].

Root canal treatment involves multiple stages, including the use of calcium hydroxide (CH) as an antibacterial dressing [6]. CH is known for its anti-endotoxin properties due to its high pH, which induces antibacterial effects [7]. However, its efficacy against microbial biofilms, especially Enterococcus faecalis, is controversial [8].

Antimicrobial photodynamic therapy (aPDT) is a minimally invasive approach used in healthcare to treat various diseases [9–13]. In endodontics, aPDT is recommended post-biomechanical preparation and pre-intracanal medication to enhance conventional therapy [5, 14]. It uses a photosensitizer (PS), light source, and oxygen to produce radicals that eradicate target microorganisms [15]. Research shows significant reductions in microorganisms like *Enterococcus faecalis* and *Porphyromonas gingivalis* [2, 14], along with decreased post-operative pain and improved periapical tissue healing [16, 17].

Selecting the appropriate PS and light source is crucial for the effectiveness of aPDT [18, 19]. Among the PSs approved for intraoral use and widely utilized in dental applications, methylene blue (MB) and toluidine blue compounds are considered non-toxic and have demonstrated proven antimicrobial action in aPDT [20]. Their cationic charge facilitates penetration of the outer membrane of gram-negative bacteria, exhibiting high affinity for bacterial cells over host cells [21]. Another safe and widely researched PS is curcumin (CC), a natural compound with known chemical and pharmacological effects [15, 22, 23]. With a broad absorption spectrum (300–500 nm), CC serves as a photochemical compound suitable for aPDT [20, 24]. Indocyanine green (IG), a synthetic fluorescent dye approved by the US Food and Drug Administration, is a promising PS option for antimicrobial photodynamic therapy (aPDT). IG exhibits strong absorption in the near-infrared spectrum, particularly within the range of 800 nm to 810 nm, allowing for efficient activation with minimal scattering interference. This property enhances its antimicrobial efficacy and enables deeper light penetration into biological tissues, making it particularly suitable for use in the complex anatomy of root canal systems [25, 26].

Recent works have highlighted the impact of various PSs and lasers on the mechanical characteristics of root dentin and the resin-based cements bonding strength [5, 20, 22, 24]. However, there is a notable gap in the literature regarding comparative studies examining the effects of PS-IG in comparison to other PSs, either alone or in combination with intracanal medication such as CH, on the bond strength of glass-fiber posts (GFPs), the chemical composition of the intraradicular surface, or the sealing of the adhesive interface. This gap is particularly significant for teeth that have undergone endodontic treatment and exhibit substantial coronal destruction, as they necessitate intraradicular retainers for functional and aesthetic restoration.

This in vitro study aimed to assess the impact of aPDT using IG, CC, and MB, with or without CH, on the push-out bond strength, adhesive interface sealing, and chemical composition of GFPs to different thirds of intraradicular dentin. The null hypotheses were: (1) Different PSs in aPDT, alone or with intracanal medication, do not yield significant differences in push-out bond strength, adhesive interface sealing of GFPs to intraradicular dentin, or root dentin chemical composition; (2) Different intraradicular thirds do not result in significant differences in bond strength, adhesive interface sealing, and chemical composition of intraradicular dentin.

## Materials and methods

### Experimental design

**Fig. 1:**
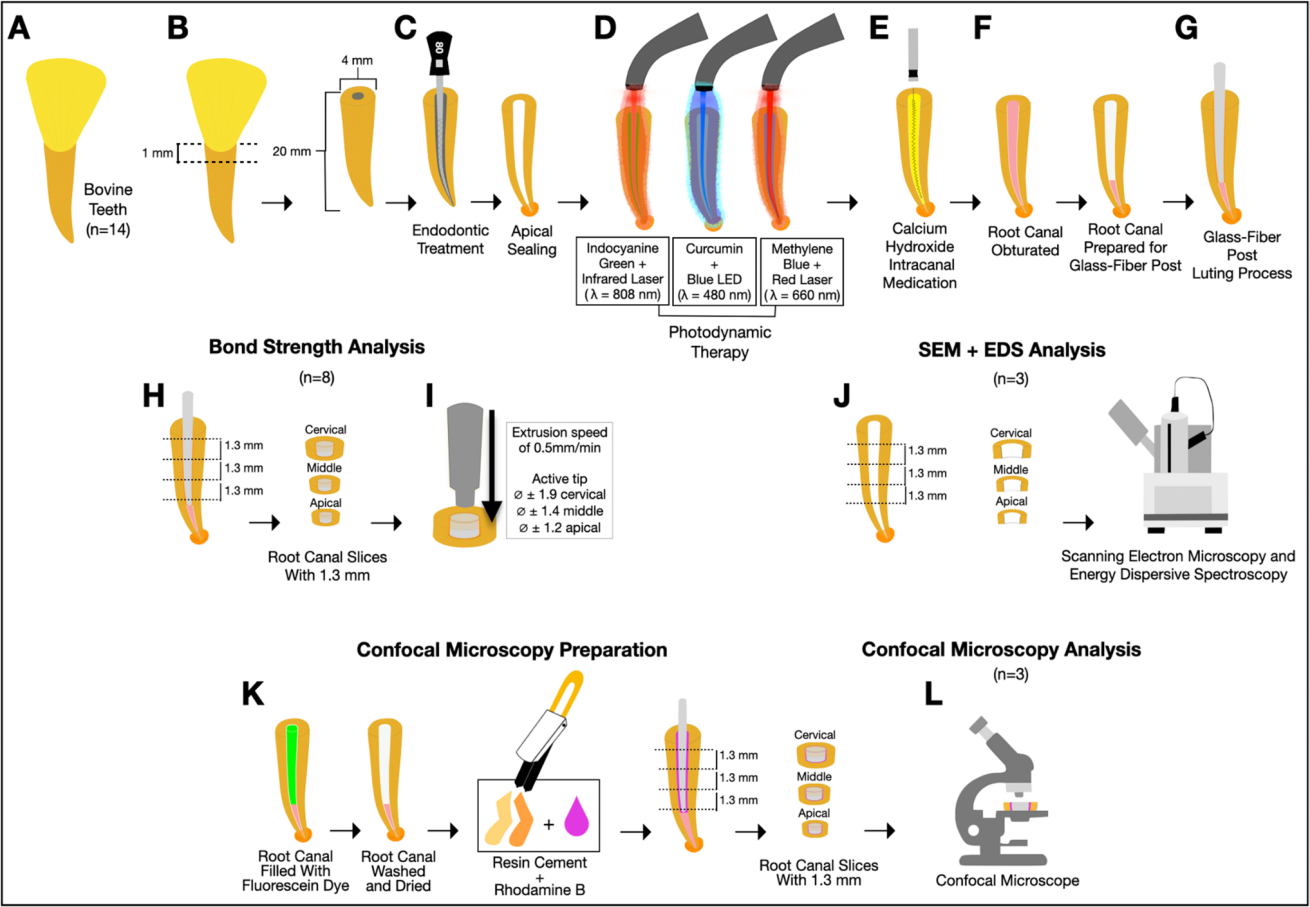
Sample preparation: **A**, Bovine teeth; **B**, Removal of anatomical crowns; **C**, Standardization of root canal length and diameter, and apical region sealing; **D**, aPDT session using indocyanine green (λ 808 nm), curcumin (λ 480 nm), and methylene blue (λ 660 nm); **E**, Calcium hydroxide intracanal medication; **F**, Samples obturation; **G**, Post-luting; **H**, Preparation of 1.3-mm thick intraradicular slices for push-out bond strength tests; **I**, Push-out bond strength test; **J**, Preparation of specimens for SEM and EDS analysis; **K**, Specimen preparation for confocal microscopy; **L**, Preparation of slices for confocal microscopy analysis

The materials used are detailed in Table 1, and the ethics approval number is #0418/2022. 120 bovine incisor teeth from approximately 3-year-old cattle, free from cracks, fractures, or curved roots, were used (Fig. 1-A) [5, 27]. The anatomical crowns were removed with a low-speed diamond saw to standardize root length at 20 mm and maintain an average root canal diameter of 4 mm (Fig. 1-B). Biomechanical root canal instrumentation was performed using manual K-Files #45 to #80 (Dentsply-Maillefer, Ballaigues, Switzerland) (Fig. 1-C). Root canals were irrigated with 10 ml of 2.5% sodium hypochlorite at each file change, followed by 17% ethylenediaminetetraacetic acid (EDTA; Biodinâmica Química e Farmacêutica LTDA, Ibiporã, PR, Brazil) for 3 min, then rinsed with 2.5% sodium hypochlorite to remove the smear layer. Apices were sealed with dental adhesive (Adper Single Bond 2; 3 M ESPE, St Paul MN, USA) and resin composite (Filtek Z250 XT, 3 M ESPE, St Paul MN, USA) to prevent PS extravasation during root canal obturation (Fig. 1-C) [28, 29].

**Table 1:** Materials, classification, composition, and batch numbers of the materials used

Material	Classification	Composition*	Batch
Adper Single Bond 2 (3M ESPE)	Adhesive System	BisGMA, HEMA, glycerol 1,3-dimethacrylate, diurethane dimethacrylate, water, ethanol, photoinitiators, 5 nm silanized silica, polyacrylic and itaconic acid copolymer.	N820206
Filtek Z350XT (3M ESPE)	Resin Composite	Bis-EMA, Bis-GMA, TEGDMA, UDMA, silica and zirconia nanofillers, and, agglomerated zirconia silica nanoclusters.	HB004209993
Calcium Hydroxide (Biodinamica)	Intracanal Medication	38% of calcium hydroxide and, 62% of barium oxide. (pH ≅ 12.4)	36870
Iodoform (Biodinamica)	Intracanal Medication	Iodoform (99–100%)	005-22
Propylene Glycol (Farma Formula)	Intracanal Medication	100% propylene glycol	530080
MTA Fillapex (Angelus)	Endodontic Sealer	Salicylate resin, natural resin, diluting resin, bismuth oxide, nanoparticulated silica, MTA and pigments.	69038
RelyX Ceramic Primer (3M ESPE)	Ceramic Primer	Ethyl alcohol, water, and 3-MPS.	2106800707
RelyX U200 (3M ESPE)	Resin Cement	Base: methacrylate phosphoric acid esters, glass-fiber, triethylene glycol dimethacrylate, sodium persulfate, silane-treated silica. Catalyst: substitute dimethacrylate, glass fiber, sodiump-toluenesulfonate, silane-treated silica, calcium.	25015

*Bis-EMA* ethoxylated bisphenol A glycol dimethacrylate, *HEMA* 2-hydroxyethyl methacrylate, *Bis-GMA* bisphenol-A diglycidyl ether dimethacrylate, *TEGDMA* triethylene glycol dimethacrylate, *UDMA* urethane dimethacrylate, *MTA* mineral trioxide aggregate,* 3-MPS* 3-methacryloxypropyl-trimethoxy silane

*According to manufacturer’s information

## Antimicrobial photodynamic therapy and experimental groups

Table 2 details the eight experimental groups (*n* = 14). The negative control group (NC) received deionized water only, while the positive control (PC) received deionized water and intracanal medication for 14 days. Groups subjected to aPDT used PS-IG 50 mg/L (Ophthalmos S/A, São Paulo, SP, Brazil) with an infrared Laser (λ = 808 nm; 100 mW; 6 J, 200 J/cm^2^, output area of 3 mm²; Laser Duo; MMO, São Carlos, SP, Brazil) for 60 s [25], PS-CC 500 mg/L (Sigma Aldrich, Merck KGaA, St. Louis, MO, US) with a 480 nm blue LED for 240 s [21, 28], and PS-MB 50 mg/L (Chimiolux, DMC, São Carlos, SP, Brazil) with a red Laser (λ = 660 nm; 100 mW; 6 J, 200 J/cm^2^, output area of 3 mm²; Laser Duo; MMO, São Carlos, SP, Brazil) for 60 s [27, 29] (Fig. 1-D). During the pre-irradiation period, PSs were agitated for one minute. An ultrasonic insert (Helse Dental Technology, Santa Rosa de Viterbo, SP, Brazil) positioned 2 mm below the working length was used with an ultrasound device (NEWTRON P5 XS, Satelec Acteon, Indaiatuba, SP, Brazil) set to power scale 2, avoiding canal wall contact [27, 28]. PS activation used a 300 μm-diameter optical fiber (DMC Equipamentos, São Carlos, SP, Brazil) with helical movements, moving apically to cervical regions at 10 times per minute to a depth 2 mm shorter than the working length [29, 30]. Post-aPDT, canals were irrigated with 10 mL deionized water, then dried with air jets and sterile paper points (Dentsply Sirona, York, PA, USA).

**Table 2:** Distribution of the experimental groups

Groups Abbreviation	Treatment Details
NC	Root canals were irrigated with deionized water (Negative control).
PC	Root canals were irrigated with deionized water and filled with calcium hydroxide-based intracanal medication (Positive control).
IG	Root canals were filled with indocyanine green photosensitizer at 50 mg/L activated by infrared Laser (λ 808 nm).
IG + CH	Root canals were filled with indocyanine green photosensitizer at 50 mg/L activated by infrared Laser (λ 808 nm) and filled with calcium hydroxide-based intracanal medication.
CC	Root canals were filled with curcumin photosensitizer at 500 mg/L activated by blue LED light (λ 480 nm).
CC + CH	Root canals were filled with curcumin photosensitizer at 500 mg/L activated by blue LED light (λ 480 nm) and filled with calcium hydroxide-based intracanal medication.
MB	Root canals were filled with methylene blue photosensitizer at 50 mg/L activated by red Laser (λ 660nm).
MB + CH	Root canals were filled with methylene blue photosensitizer at 50 mg/L activated by red Laser (λ 660nm) and filled with calcium hydroxide-based intracanal medication.

## Calcium hydroxide medication

Groups PC, IG + CH, CC + CH, and MB + CH received CH intracanal medication mixed with iodoform (Biodinâmica Química e Farmacêutica, Ibiporã, PR, Brazil) and propylene glycol (Farma Formula, Contagem, MG, Brazil), introduced using a #4 Lentulo instrument (Fig. 1-E). Samples were sealed with interim cement and stored in 100% humidity at 37 °C for 14 days. Radiographs confirmed CH filling. After this period, the coronal seal and CH were removed with 2.5% sodium hypochlorite and an endodontic file (Maillefer Instruments - K#80, Tulsa, OK, USA). Another radiographic analysis confirmed complete CH removal [5].

## Bonding strength analysis (push-out)

Samples were loaded with MTA Fillapex sealer (Angelus, Londrina, PR, Brazil), gutta-percha cones (#80), and F and MF auxiliary cones (Dentsply Sirona, York, PA, USA) (*n* = 8)(Fig. 1-F). The obturation used Tagger’s technique. Teeth were stored in 100% humidity at 37 °C [21, 29]. After a week, gutta-percha up to 9 mm depth was removed with a #2 White Post DCE system drill (White Post DCE #2; FGM, Joinville, SC, Brazil). Canals were flushed with 10 ml of 0.9% physiological saline, dried with sterile paper points. GFPs were etched with 37% phosphoric acid (FGM, Joinville, SC, Brazil) for 60 s, treated with silane RelyX Ceramic Primer (3 M ESPE, St Paul MN, USA) for 60 s, and dried with gentle air to prevent surface contamination. The RelyX U200 resin-based cement (3 M ESPE, St Paul MN, USA) was dispensed with a mixing tip and injected into the post space (Fig. 1-G). The resin cement was evenly spread onto the GFP surface, then the intraradicular retainer was placed into the canal. Polymerization was performed for 40 s from the cervical area with an LED light curing device (Radii-Cal; SDI, Bayswater, Australia) at 1200 mW/cm². Samples were maintained for 7 days at 37 °C and 100% humidity [24, 31].

Specimens were sectioned perpendicularly into 1.3 mm thick slices (cervical, middle, and apical) (Fig. 1-H). Slice thickness and internal base diameters were measured using a digital caliper (Mitutoyo, Aurora, IL, USA) [31]. The push-out test was performed on a universal testing machine (DL3000, EMIC, São José dos Pinhais, PR, Brazil). A metal tip with specific diameters for each root third (cervical third − 1.90 mm; middle third − 1.40 mm; and apical third − 1.20 mm) was fixed at the upper portion, while the sample was secured on a stainless steel support at the lower portion. Compressive load was applied at 0.5 mm/min (Fig. 1-I), and push-out bond strength values were calculated as [31]:

$$\mathrm{POBS}=\mathrm F/\mathrm A$$

where POBD represents the push-out bond strength (MPa); F is the maximum force (N); and A is the adhesive interface area (mm^2^) calculated using the following equation [31]:

$$A=\pi\left(R_C+R_A\right)\sqrt{\left(R_C-R_A\right)^2+h^2}$$

where π is 3.14; R_C_ and R_A_ are the largest (coronal) and smallest (apical) post radii, respectively; and h is the height of the third slice.

Post-test, slices were bisected to examine the adhesive interface using a stereomicroscope (Stemi SV11, Zeiss, NY, USA) at ×6 and ×66 magnifications, identifying failure modes: mixed, adhesive, cohesive in dentin, and cohesive in resin cement. Representative samples were gold-coated and examined using SEM (JEOL, 5600LV- JSM, Tokyo, Japan) to visualize fracture patterns [24].

## Substrate morphology and chemical composition analysis

After CH removal, twenty-four samples (*n* = 3) were sectioned into 1.3 mm slices representing the cervical, middle, and apical thirds. These were analyzed using SEM with an energy dispersive spectroscopy (EDS) system. EDS assessed the relative abundance of carbon (C), oxygen (O), phosphorus (P), calcium (Ca), magnesium (Mg), sodium (Na), silicon (Si), and zinc (Zn). Five positions per specimen were pre-selected and analyzed to determine chemical composition, with an arithmetic mean calculated for each third (Fig. 1-J) [32, 33].

### Adhesive interface sealing analysis

Twenty-four specimens (*n* = 3), restored with GFPs, were examined using confocal fluorescence microscopy. Two dyes were used for visualization: fluorescein (0.1%) (FDA, Sigma Chemical Co, St. Louis, MO, USA), introduced into the root canals after removing 9 mm of the filling material, and Rhodamine B (Sigma Chemical, USA), incorporated into the resin cement at 0.16 µg/g before post-luting (Fig. 1-K). Specimens were stored in 100% humidity at 37 °C for 7 days. Slices were examined using a confocal laser scanning microscope (CLSM)(Leica TCS SP2; Leica Microsystems, Heidelberg, Germany). The excitation used an argon laser (488 nm) and a He-Ne laser (453 nm) [34]. Confocal micrographs were captured in fluorescent mode using an oil immersion objective lens (×40, numerical aperture 1.25). Photos of four regions per sample were taken and assembled for analysis. Interface parameters (dentin/cement adhesive interface quality, formation of tags in intraradicular dentin, and tag penetration depth) were evaluated based on scores by Strazzi-Sahyon et al., 2023 [5] and Moda et al., 2018 [35].

### Statistical analysis

Bond strength, chemical composition, and adhesive interface sealing data underwent normality (Shapiro-Wilk) and homogeneity (Bartlett) tests. Push-out bond strength and chemical composition data were analyzed using two-way repeated measures Analysis of Variance (ANOVA) followed by Tukey’s post hoc test (α = 0.05) (Minitab Statistical Software, version 19.2020.1.0). The evaluation of adhesive interface sealing was conducted through inter-examiner Kappa testing and subjected to analysis via Kruskal-Wallis and Dunn’s tests (α = 0.05) using SigmaPlot Version 12.0.

## Results

### Push-out bond strength

The push-out bond strength values are summarized in Table 3. Analysis revealed significant differences in bond strength among different root thirds within the NC group, with lower values in the apical third compared to the cervical and middle thirds (*P* < .05). The IG group showed higher values in the apical third relative to its cervical region (*P* = .007), while the IG + CH group had elevated values in the cervical third compared to its middle third (*P* = .015).

**Table 3:** Mean ± standard deviation values of push-out bond strength (MPa) of intraradicular dentin as function of the experimental groups and intraradicular thirds

Groups	NC	PC	IG	IG + CH	CC	CC + CH	MB	MB + CH
Thirds								
Cervical	5.37 ± 2.36 Aabc	3.93 ± 1.17 Abcd	2.64 ± 1.53 Bd	6.49 ± 2.92 Aa	4.18 ± 1.40 Abcd	6.03 ± 2.12 Aab	4.54 ± 2.02 Aabcd	3.44 ± 1.65 Acd
Middle	5.59 ± 4.21 Aa	3.10 ± 2.11 Ab	3.45 ± 2.11 ABab	3.50 ± 2.48 Bab	4.94 ± 1.80 Aab	4.95 ± 2.59 Aab	4.72 ± 2.84 Aab	4.14 ± 1.37 Aab
Apical	3.04 ± 1.26 Bb	3.36 ± 1.69 Ab	5.67 ± 2.42 Aa	4.51 ± 2.73 ABab	6.14 ± 1.66 Aa	4.56 ± 2.59 Aab	5.57 ± 2.53 Aa	4.02 ± 2.15 Aab

Different letters, uppercase in column and lowercase in row, indicate statistically significant differences (*P*<.05)

*NC* negative control (root canals irrigated with deionized water), *PC* positive control (root canals irrigated with deionized water and filled with calcium hydroxide-based intracanal medication), *IG* root canals were filled with indocyanine green photosensitizer at 50 mg/L activated by infrared Laser (λ 808 nm), *IG + CH* root canals were filled with indocyanine green photosensitizer at 50 mg/L activated by infrared Laser (λ 808 nm) and filled with calcium hydroxide-based intracanal medication, *CC* root canals were filled with curcumin photosensitizer at 500 mg/L activated by blue LED light (λ 480 nm), CC + CH: root canals were filled with curcumin photosensitizer at 500 mg/L activated by blue LED light (λ 480 nm) and filled with intracanal calcium hydroxide-based intracanal medication, *MB* root canals were filled with methylene blue photosensitizer at 50 mg/L activated by red Laser (λ 660nm), *MB + CH* root canals were filled with methylene blue photosensitizer at 50 mg/L activated by red Laser (λ 660nm) and filled with calcium hydroxide-based intracanal medication

In the cervical third, the IG + CH group had significantly higher bond strength compared to the PC, IG, CC, and MB + CH groups (*P* < .05). Conversely, the IG group had the lowest bond strength in the cervical third, significantly differing from the NC, IG + CH, and CC + CH groups (*P* < .05). Additionally, the MB + CH group had lower bond strength than the CC + CH group in the cervical third (*P* = .021). For the middle third, the only significant difference was between the NC and PC groups, with the NC group showing higher values (*P* = .027). In the apical third, both the NC and PC groups had lower bond strength compared to the IG, CC, and MB groups (*P* < .05).

**Fig. 2:**
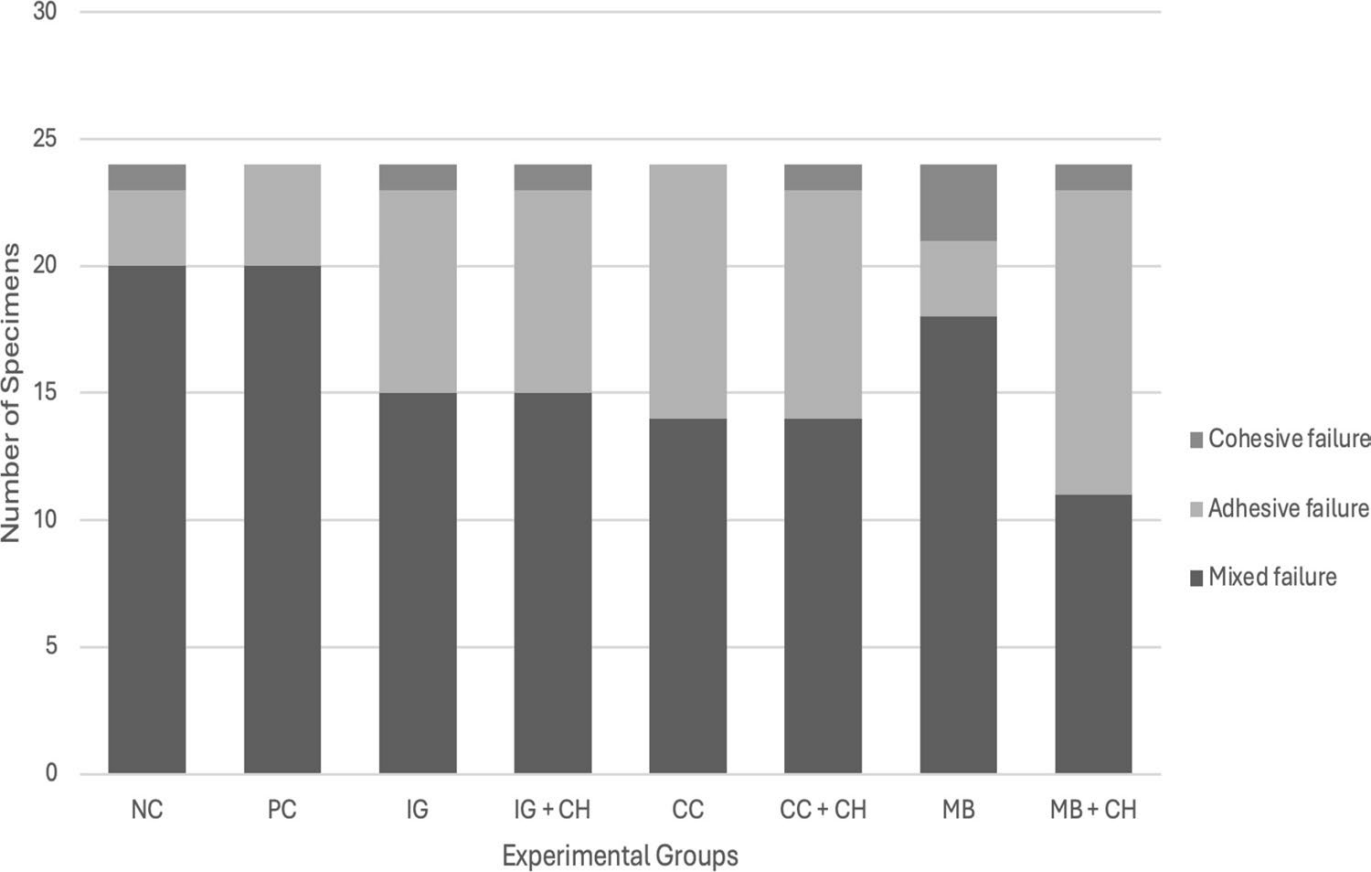
Occurrence of failure patterns of the specimens (number of sample thirds)

**Fig. 3:**
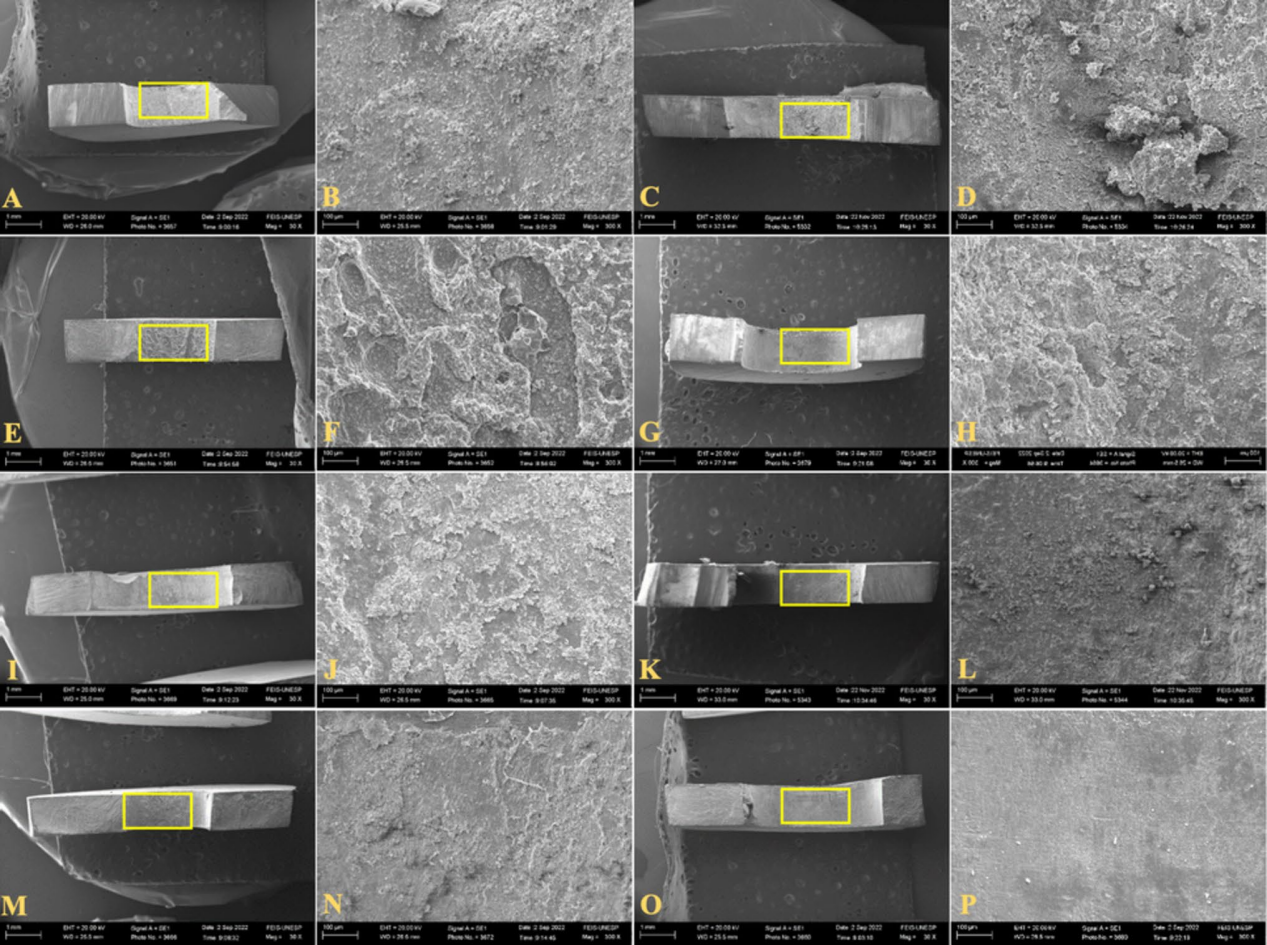
Scanning electron microscopy images with original magnification of ×30 and ×300, respectively. **A**, **B** – Mixed-type failure of NC group; **C**,**D** - Mixed-type failure of PC group; **E**,**F** - Mixed-type failure of IG group; **G**,**H** - Mixed-type failure of IG + CH group; **I**,**J** - Mixed-type failure of CC group; **K**,**L** – Mixed-type failure of CC + CH group; M,N - Mixed-type failure of MB group; O,P - Adhesive-type failure of MB + CH group

Failure type analysis revealed a predominance of mixed failures across all groups, except for the MB + CH group, where adhesive failure predominated (Fig. 2). Figure 3 shows representative SEM images illustrating the failure patterns observed in each group.

### Substrate morphology and chemical composition

**Fig. 4:**
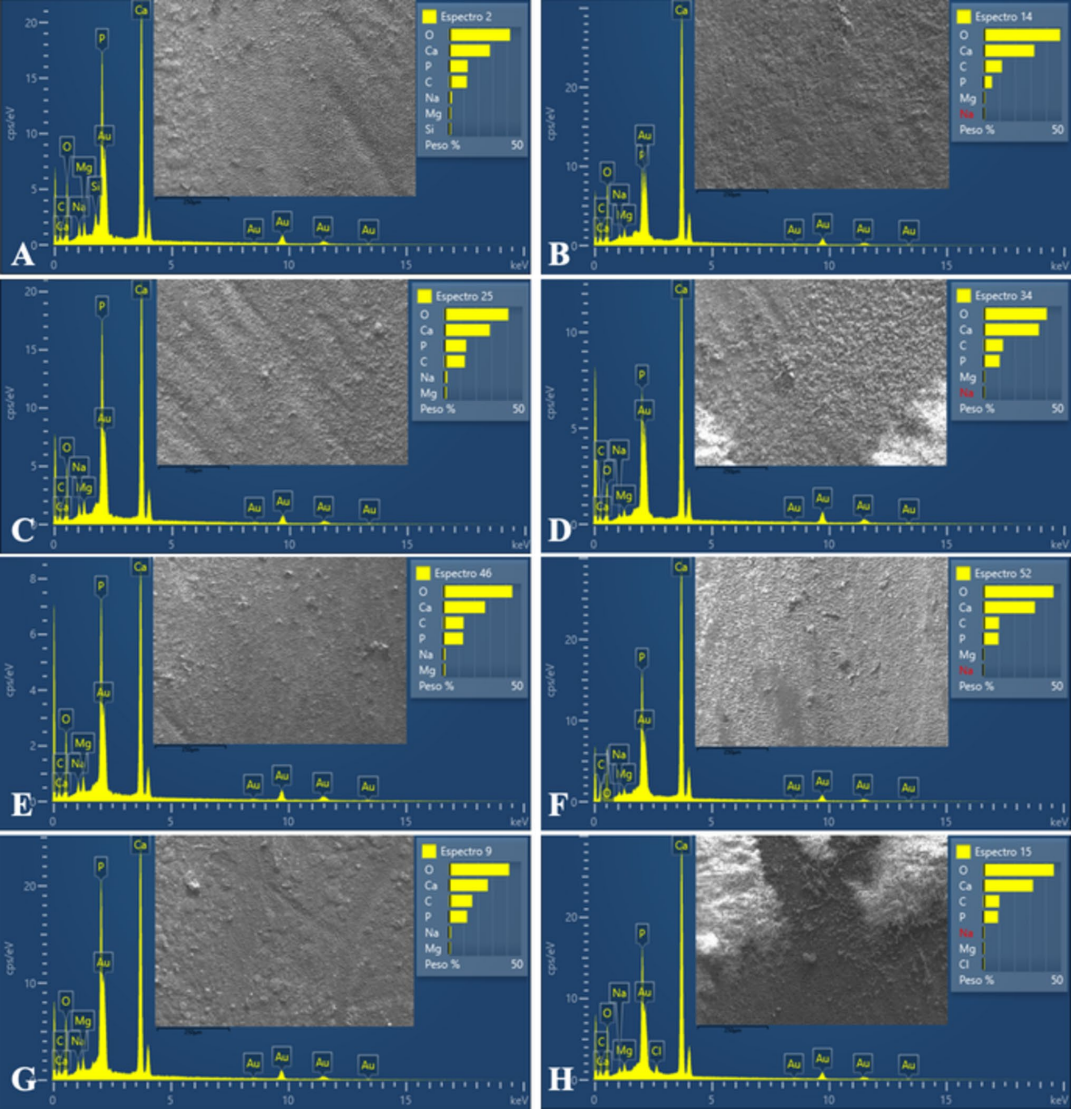
Energy-dispersive X-ray spectra of intraradicular dentin surface and scanning electron micrographs under original magnification (×300), according to each experimental group. A – NC group; B – PC group; C – IG group; D - IG + CH group; E – CC group; F - CC + CH group; G – MB group; H - MB + CH group

Figure 4 presents the energy-dispersive X-ray spectra of the intraradicular dentin surface alongside SEM micrographs corresponding to each experimental group. Table 4 outlines the percentage values representing the mass of chemical elements identified on the dentin surface for each group and root third.

**Table 4:** Mean ± standard deviation values of the root dentin chemical element content (%) as function of the experimental groups and intraradicular thirds

Groups	NC	PC	IG	IG + CH	CC	CC + CH	MB	MB + CH
Thirds								
				**Carbon (C)**				
Cervical	10.10 ± 2.61 Aa	9.58 ± 0.59 Aa	12.98 ± 0.13 Aa	11.96 ± 0.81 Aa	10.51 ± 2.12 Aa	10.45 ± 0.66 Aa	12.38 ± 0.87 Aa	11.27 ± 0.69 Aa
Middle	11.34 ± 2.41 Aa	10.87 ± 0.97 Aa	13.45 ± 1.23 Aa	11.70 ± 0.73 Aa	17.57 ± 6.05 Aa	10.82 ± 1.49 Aa	13.44 ± 1.18 Aa	13.18 ± 1.61 Aa
Apical	12.61 ± 2.62 Aa	10.44 ± 0.80 Aa	13.58 ± 0.038 Aa	11.16 ± 2.93 Aa	12.78 ± 0.65 Aa	10.88 ± 2.23 Aa	14.36 ± 1.58 Aa	12.30 ± 1.76 Aa
				**Oxygen (O)**				
Cervical	40. 82 ± 2.71 Aa	44.78 ± 3.02 Aa	42.47 ± 1.44 Aa	43.14 ± 3.11 Aa	41.64 ± 2.18 Aa	45.45 ± 1.32 Aa	39.47 ± 0.23 Ba	41.84 ± 1.28 Ba
Middle	42.34 ± 2.34 Aa	43.94 ± 6.83 Aa	43.00 ± 0.53 Aa	43.78 ± 1.07 Aa	41.57 ± 1.79 Aa	43.77 ± 0.92 Aa	41.63 ± 0.52 Aa	43.65 ± 0.72 ABa
Apical	40.51 ± 3.30 Ac	46.31 ± 1.46 Aa	43.02 ± 0.99 Aabc	45.30 ± 0.88 Aa	43.08 ± 1.04 Aabc	44.84 ± 0.24 Aab	40. 72 ± 0.87 ABbc	45.00 ± 0.93 Aa
				**Phosphorus (P)**				
Cervical	14.13 ± 2.21 Aa	6.68 ± 0.60 Ac	13.47 ± 0.49 Aab	9.59 ± 2.24 Abc	14.08 ± 1.56 Aa	10.30 ± 1,96Aabc	13.58 ± 1.37 Aab	10.91 ± 1.67 Aab
Middle	13.56 ± 2.03 Aa	5.17 ± 0.14 Bb	13.36 ± 0.37 Aa	9.70 ± 1.81 Aab	12.37 ± 2.21 Aa	9.70 ± 1.96 Aab	13.33 ± 0.50 Aa	9.89 ± 2.53 Aab
Apical	13.67 ± 1.73 Aa	6.80 ± 0.57 Ac	13.17 ± 0.31 Aab	9.98 ± 1.90 Aabc	13.31 ± 0.64 Aa	9.09 ± 1.93 Abc	12.75 ± 0.94 Aab	9.89 ± 2.38 Aabc
				**Calcium (Ca) **				
Cervical	31.56 ± 3.41 Aab	36.62 ± 1.55 Aa	28.21 ± 0.92 Ab	33.05 ± 1.99 Aab	31.00 ± 2.19 Aab	31.97 ± 2.08 Aab	32.28 ± 0.95 Aab	33.63 ± 1.76 Aab
Middle	29.38 ± 2.67 Aa	30.56 ± 5.68 Aa	27.71 ± 0.06 Aa	32.69 ± 1.42 Aa	25.92 ± 5.52 Aa	34.01 ± 2.44 Aa	29.35 ± 1.59 Aa	31.56 ± 1.16 Aa
Apical	30.02 ± 2.53 Aab	33.89 ± 1.62 Aa	27.80 ± 1.27 Ab	31.32 ± 1.55 Aab	27.91 ± 0.89 Ab	32.48 ± 2.26 Aab	29.83 ± 2.68 Aab	30.86 ± 2.48 Aab
				**Magnesium (Mg) **				
Cervical	1.28 ± 0.23 Aab	0.79 ± 0.04 Ac	1.22 ± 0.27 Aabc	1.05 ± 0.14 Aabc	1.38 ± 0.21 Aa	0.82 ± 0.06 Abc	1.05 ± 0.16 Aabc	1.04 ± 0.16 Aabc
Middle	1.29 ± 0.22 Aa	0.91 ± 0.18 Aa	1.18 ± 0.30 Aa	1.23 ± 0.10 Aa	1.26 ± 0.12 Aa	0.88 ± 0.09 Aa	1.04 ± 0.09 Aa	0.97 ± 0.16 Aa
Apical	1.19 ± 0.22 Aa	0.95 ± 0.13 Aa	1.17 ± 0.28 Aa	1.09 ± 0.09 Aa	1.45 ± 0.12 Aa	0.93 ± 0.32 Aa	1.02 ± 0.16 Aa	0.90 ± 0.25 Aa
				**Sodium (Na)**				
Cervical	1.73 ± 0.13 Aa	0.87 ± 0.06 Ab	1.26 ± 0.49 Aab	0.88 ± 0.24 Ab	1.38 ± 0.12 Aab	1.01 ± 0.17 Ab	1.23 ± 0.26 Aab	0.85 ± 0.03 Ab
Middle	1.72 ± 0.16 Aa	1.43 ± 1.19 Aa	1.30 ± 0.32 Aa	0.90 ± 0.06 Aa	1.15 ± 0.21 Aa	0.84 ± 0.14 Aa	1.19 ± 0.26 Aa	0.74 ± 0.17 Aa
Apical	1.63 ± 0.24 Aa	0.85 ± 0.11 Acd	1.26 ± 0.29 Aabcd	0.99 ± 0.18 Abcd	1.47 ± 0.16 Aab	0.78 ± 0.05 Ad	1.32 ± 0.10 Aabc	0.76 ± 0.21 Ad
				**Silicon (Si)**				
Cervical	0.37 ± 0.65 Aa	0.00 ± 0.00 Aa	0.00 ± 0.00 Aa	0.00 ± 0.00 Aa	0.00 ± 0.00 Aa	0.00 ± 0.00 Aa	0.00 ± 0.00 Aa	0.00 ± 0.00 Aa
Middle	0.38 ± 0.65 Aa	0.00 ± 0.00 Aa	0.00 ± 0.00 Aa	0.00 ± 0.00 Aa	0.16 ± 0.28 Aa	0.00 ± 0.00 Aa	0.00 ± 0.00 Aa	0.00 ± 0.00 Aa
Apical	0.36 ± 0.63 Aa	0.00 ± 0.00 Aa	0.00 ± 0.00 Aa	0.00 ± 0.00 Aa	0.00 ± 0.00 Aa	0.00 ± 0.00 Aa	0.00 ± 0.00 Aa	0.00 ± 0.00 Aa
				**Zinc (Zn)**				
Cervical	0.00 ± 0.00 Aa	0.50 ± 0.87 Aa	0.00 ± 0.00 Aa	0.33 ± 0.57 Aa	0.00 ± 0.00 Aa	0.00 ± 0.00 Aa	0.00 ± 0.00 Aa	0.07 ± 0.12 Aa
Middle	0.00 ± 0.00 Aa	6.98 ± 12.08 Aa	0.00 ± 0.00 Aa	0.00 ± 0.00 Aa	0.00 ± 0.00 Aa	0.00 ± 0.00 Aa	0.00 ± 0.00 Aa	0.00 ± 0.00 Aa
Apical	0.00 ± 0.00 Aa	0.76 ± 1.32 Aa	0.00 ± 0.00 Aa	0.00 ± 0.00 Aa	0.00 ± 0.00 Aa	1.01 ± 1.74 Aa	0.00 ± 0.00 Aa	0.00 ± 0.00 Aa

Different letters, uppercase in column and lowercase in row, indicate statistically significant differences for each chemical element (*P*<.05)

*NC* negative control (root canals irrigated with deionized water), *PC* positive control (root canals irrigated with deionized water and filled with calcium hydroxide-based intracanal medication), *IG* root canals were filled with indocyanine green photosensitizer at 50 mg/L activated by infrared Laser (λ 808 nm), *IG + CH* root canals were filled with indocyanine green photosensitizer at 50 mg/L activated by infrared Laser (λ 808 nm) and filled with calcium hydroxide-based intracanal medication, *CC* root canals were filled with curcumin photosensitizer at 500 mg/L activated by blue LED light (λ 480 nm), *CC + CH* root canals were filled with curcumin photosensitizer at 500 mg/L activated by blue LED light (λ 480 nm) and filled with intracanal calcium hydroxide-based intracanal medication, *MB* root canals were filled with methylene blue photosensitizer at 50 mg/L activated by red Laser (λ 660nm), *MB + CH* root canals were filled with methylene blue photosensitizer at 50 mg/L activated by red Laser (λ 660nm) and filled with calcium hydroxide-based intracanal medication

Oxygen levels in the apical third were significantly lower in the NC group compared to the PC, IG + CH, CC + CH, and MB + CH groups (*P* < .05). The PC, IG + CH, and MB + CH groups had higher oxygen levels in the apical third compared to the NC and MB groups (*P* < .05). Within the MB group, oxygen levels were lower in the cervical third compared to the middle region (*P* = .013), while the MB + CH group had higher levels in the apical third compared to the cervical third (*P* = .022).

For phosphorus levels, in the cervical third, the NC and CC groups had higher values compared to the PC and IG + CH groups (*P* < .05). The PC group had lower values compared to the NC, IG, CC, MB, and MB + CH groups (*P* < .05). In the middle region, the PC group had lower values compared to the NC, IG, CC, and MB groups (*P* < .05). In the apical third, the NC and CC groups had higher values compared to the PC and CC + CH groups (*P* < .05). Only the PC group showed a disparity in phosphorus levels, with lower values in the middle third compared to the apical and cervical regions (*P* < .05).

For calcium levels, the PC group had higher values in the cervical and apical thirds compared to the IG group (*P* < .05). In the apical third, the PC group had higher values compared to the IG and CC groups (*P* < .05). No significant differences were observed between the root thirds for calcium (*P* > .05).

For magnesium, the PC group had lower values than the NC and CC groups in the cervical third (*P* < .05). The CC group had higher values in the cervical region compared to the PC and CC + CH groups. No significant differences were observed between the thirds for magnesium (*P* > .05).

In the sodium analysis, the NC group had higher values than the PC, IG + CH, CC + CH, and MB + CH groups in the cervical third (*P* < .05). In the apical third, the NC and CC groups had higher values compared to the PC, CC + CH, and MB + CH groups (*P* < .05). No significant differences were observed between the thirds for sodium (*P* > .05).

No significant differences were detected across groups and root thirds for carbon, silicon, and zinc (*P* > .05). Silicon was detected only in the NC and CC groups, while zinc was found in some thirds of the groups with CH medication, despite the lack of significant differences.

### Adhesive interface sealing

**Fig. 5:**
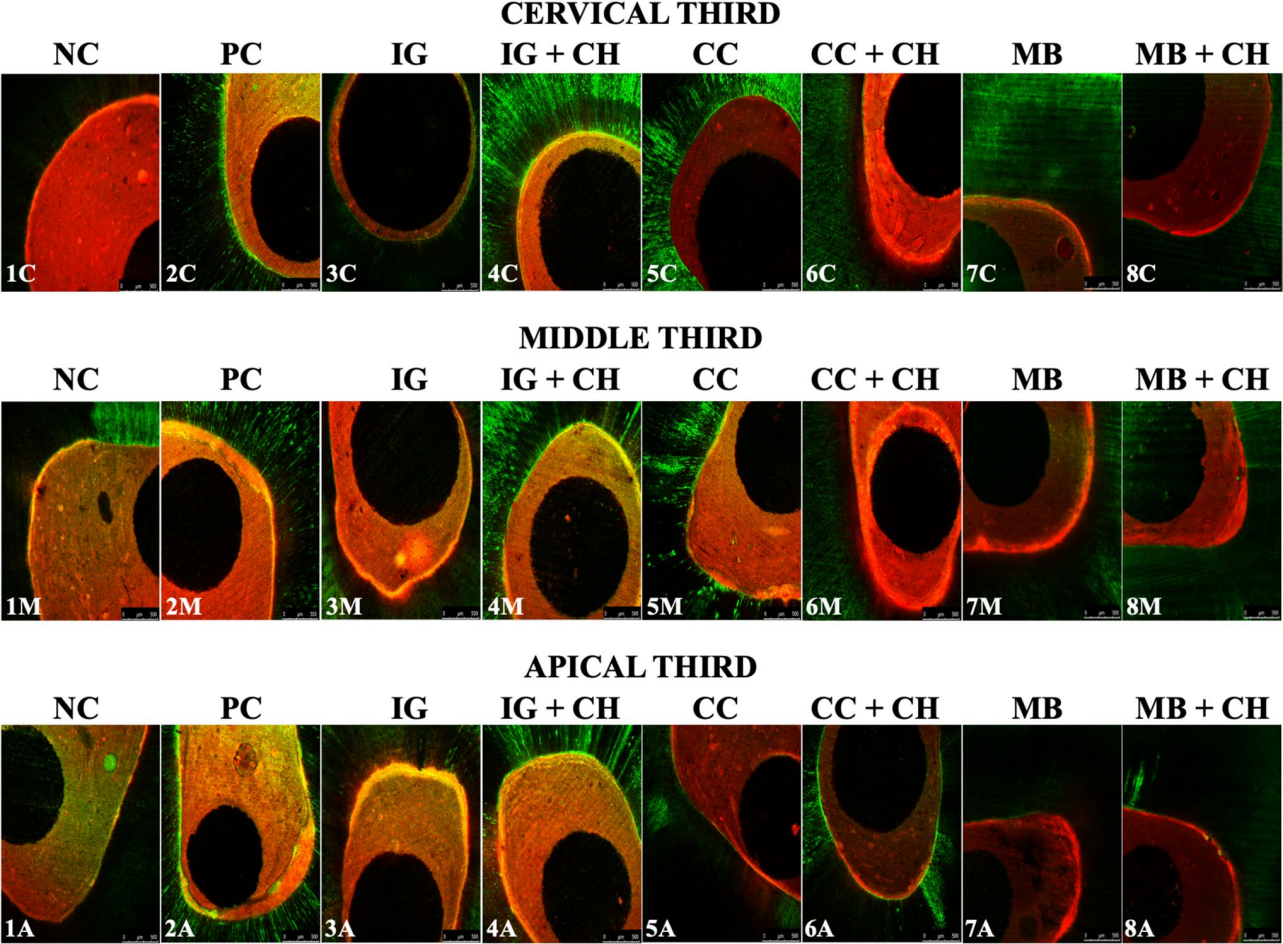
Confocal laser scanning micrographs of the adhesive interface sealing according to the experimental groups and intraradicular thirds under original magnification ×5

The results of the adhesive interface sealing assessment, including scores and micrographs obtained via confocal laser scanning microscopy, are presented in Table 5; Fig. 5, respectively. Inter-examiner agreement scores were 0.78, 0.84, and 0.80 for interface quality, tag formation, and tag penetration depth, respectively. No significant differences were observed among experimental groups or root thirds for any criteria (*P* > .05) (Table 5). Score 0 predominated in adhesive interface quality across all groups, except for the CC + CH, MB, and MB + CH groups, where score 1 was more common. For tag formation in dentin, scores 1 and 2 predominated. Tag penetration depth was primarily score 2 in all groups, except for the CC + CH group, where score 1 was more prevalent.

**Table 5:** Scores of adhesive interface sealing based on experimental groups and intraradicular thirds

Parameters	Quality of the dentin/cement adhesive interface				Formation of tags in intraradicular dentin					Tag penetration depth				
**Score**	0	1	2		0	1	2	3		0	1	2	3	
Grups (Thirds)														
NC														
Cervical	3	-	-	Aa	-	3	-	-	Aa	-	1	2	-	Aa
Middle	2	1	-	Aa	-	3	-	-	Aa	-	-	3	-	Aa
Apical	-	3	-	Aa	-	3	-	-	Aa	-	-	3	-	Aa
PC														
Cervical	2	1	-	Aa	-	2	1	-	Aa	-	-	3	-	Aa
Middle	1	2	-	Aa	-	2	1	-	Aa	-	-	2	1	Aa
Apical	-	3	-	Aa	-	1	2	-	Aa	-	-	3	-	Aa
IG														
Cervical	3	-	-	Aa	-	2	1	-	Aa	-	1	2	-	Aa
Middle	2	1	-	Aa	-	2	1	-	Aa	-	-	2	1	Aa
Apical	3	-	-	Aa	-	2	-	1	Aa	-	1	1	1	Aa
IG + CH														
Cervical	3	-	-	Aa	-	2	1	-	Aa	-	1	2	-	Aa
Middle	2	1	-	Aa	-	1	2	-	Aa	-	2	1	-	Aa
Apical	3	-	-	Aa	-	1	2	-	Aa	-	1	2	-	Aa
CC														
Cervical	2	1	-	Aa	-	2	1	-	Aa	-	1	1	1	Aa
Middle	2	1	-	Aa	-	1	2	-	Aa	-	-	3	-	Aa
Apical	2	1	-	Aa	-	2	1	-	Aa	5	-	3	-	Aa
CC + CH														
Cervical	2	1	-	Aa	-	2	1	-	Aa	-	2	1	-	Aa
Middle	1	2	-	Aa	-	2	1	-	Aa	-	2	1	-	Aa
Apical	1	2	-	Aa	-	1	2	-	Aa	-	3	-	-	Aa
MB														
Cervical	1	2	-	Aa	-	1	2	-	Aa	-	-	3	-	Aa
Middle	1	2	-	Aa	-	-	3	-	Aa	-	-	3	-	Aa
Apical	1	2	-	Aa	-	-	3	-	Aa	-	-	3	-	Aa
MB + CH														
Cervical	1	2	-	Aa	-	2	1	-	Aa	-	2	1	-	Aa
Middle	-	3	-	Aa	-	1	2	-	Aa	-	1	2	-	Aa
Apical	-	3	-	Aa	-	2	1	-	Aa	-	1	2	-	Aa

Different letters, uppercase in column and lowercase in row, indicate statistically significant differences for each adhesive interface parameter (*P*<.05)

*NC* negative control (root canals irrigated with deionized water), *PC *positive control (root canals irrigated with deionized water and filled with calcium hydroxide-based intracanal medication), *IG* root canals were filled with indocyanine green photosensitizer at 50 mg/L activated by infrared Laser (λ 808 nm), *IG + CH* root canals were filled with indocyanine green photosensitizer at 50 mg/L activated by infrared Laser (λ 808 nm) and filled with calcium hydroxide-based intracanal medication, *CC* root canals were filled with curcumin photosensitizer at 500 mg/L activated by blue LED light (λ 480 nm), *CC + CH* root canals were filled with curcumin photosensitizer at 500 mg/L activated by blue LED light (λ 480 nm) and filled with intracanal calcium hydroxide-based intracanal medication, *MB* root canals were filled with methylene blue photosensitizer at 50 mg/L activated by red Laser (λ 660nm), *MB + CH* root canals were filled with methylene blue photosensitizer at 50 mg/L activated by red Laser (λ 660nm) and filled with calcium hydroxide-based intracanal medication

## Discussion

The application of aPDT using different PSs with intracanal medication elicited significant variations in the push-out bond strength of GFPs and the chemical composition of intraradicular dentin. As a result, the initial hypotheses posited in this study were invalidated. Furthermore, the differing depths of root dentin, treated with aPDT and CH intracanal medication, exerted distinct influences on both the push-out bond strength and chemical composition of root dentin, leading to the rejection of the second null hypothesis.

PS-IG administration resulted in significantly reduced bond strength in the cervical third. Recent researches suggest that aPDT using PS-IG operates via a distinct mechanism compared to other PSs, involving a photothermal effect rather than the typical photochemical process [36, 37]. This effect, combined with PS-IG’s anionic nature, forms associations with plasma proteins under infrared wavelengths, hindering effective resin cement adhesion to root dentin [26]. Temperature fluctuations from PS-IG photoactivation further impact dentin’s physical and chemical properties, potentially leading to reduced adhesion [36]. Ballal et al. noted that reduced surface calcium decreases adhesive material bond strength [38]. IG, an anionic compound, can cause dentin demineralization, exacerbated by temperature increases. This effect, more pronounced in the calcium-rich cervical third, was confirmed by EDS analysis showing reduced calcium concentrations in the IG group.

In contrast to previous studies [20, 39], which observed a decline in bond strength from cervical to apical thirds, this study found higher push-out bond strengths in the apical third of the IG group compared to the cervical region. This disparity was attributed to PS distribution dynamics within the root canal; the higher PS concentration in the cervical third precipitates deposition in the middle and apical thirds, reducing adverse effects on bond strength in these regions [21, 24, 31].

PS-CC exerted a significant positive impact on GFPs’ bond strength to root dentin across all thirds, regardless of CH intracanal medication. Previous studies consistently reported enhanced bond strength values with aPDT using PS-CC due to its anionic nature, facilitating calcium ion binding and substrate porosity enhancement for effective resin cement retention [22, 28]. Investigating the interaction strengths between PSs CC and IG is crucial for understanding potential substrate damage and optimizing dentin properties through hardness and modulus of elasticity tests. Like PS-IG, PS-CC’s anionic nature binds to calcium ions in dentin, enhancing porosity and resin cement retention [33]. Investigating PS-CC and PS-IG interactions with dentin is crucial to understand potential damage. Hardness and elasticity tests could reveal insights into dentin weakening.

CC’s hydrophobic polyphenolic properties enhance adhesion by repelling moisture [28, 39]. However, research by Strazzi-Sahyon et al. showed that using PS-CC in two sessions—post-biomechanical preparation and pre-GFP luting—diminished push-out bond strength values compared to PS-MB [24].

In this study, no differences in push-out bond strength were observed between the MB group and IG and CC groups across all root thirds. However, when combined with CH intracanal medication, MB exhibited lower bond strengths in the cervical third compared to PSs associated with CH. MB’s cationic charge interacts with anionic molecules in hydroxyapatite crystals, potentially altering the calcium-phosphorus ratio and forming phosphate precipitates that obstruct resinous material interaction with root dentin [22, 36]. MB’s hydrophilic nature may also contribute to increased water absorption within dentin [31], potentially explaining higher adhesive failure rates in the MB + CH group.

The study investigated PS impacts in the presence or absence of CH intracanal medication, highlighting CH’s role in endodontic therapy for dentin repair and mineralized tissue formation [40–42]. Chemical analysis confirmed elevated calcium levels in CH-treated groups, supporting adhesion theories dependent on residual Ca^++^ presence [38]. Methods like ultrasonic irrigation for root canal cleaning were considered promising [40, 43], although their inefficacy in completely removing intracanal medication was demonstrated [44], potentially reducing resinous tag formation and adhesion effectiveness.

Groups receiving CH medication exhibited reduced phosphorus levels, significant in hydroxyapatite composition, potentially disrupting adhesion processes by obstructing dentinal tubules and altering surface chemistry [35, 38]. EDS and SEM analysis confirmed remnants of CH intracanal medication, influencing adhesive interface parameters and hindering resin monomer infiltration into dentinal tubules [6]. Elevated oxygen levels in CH-treated groups indicated enhanced antimicrobial activity and reactive oxygen species production, affecting the chemical environment crucial for adhesive interactions [45]. Elevated oxygen levels in CH-treated groups support these findings. Abu Zeid et al. noted that MTA Fillapex sealers release calcium and silicon [46], explaining the presence of silicon in the NC group and the middle third of the CC group.

CLSM examined resin-based cement interactions with root dentin, evaluating micropermeability and resin monomer infiltration, offering minimal artifact interference compared to SEM. Confocal scores and micrographs showed no significant differences in adhesive interface parameters [5]. Residual CH medication and tubule penetration depth measurement methods may have influenced the results [5].

This study’s findings underscore the significant impact of PS selection on GFPs’ bond strength to intraradicular dentin during luting procedures, emphasizing the need for personalized treatment strategies in prosthetic restoration of endodontically treated teeth. Overall, the use of laser-activated 500 mg/L curcumin in combination with CH demonstrates clinical relevance as an effective option for decontaminating root canals after biomechanical shaping and prior to the GFPs luting procedure. This approach provides antibacterial efficacy while preserving both the bonding and sealing integrity of the interface, as well as the chemical composition of the intraradicular dentin.

This study presents certain limitations, including the challenges associated with instrumentation in deeper regions of root canals, uncertainties regarding the complete removal of intracanal medication containing calcium hydroxide, and the utilization of teeth with a heterogeneous substrate. Given the in vitro nature of this research, it cannot precisely replicate the dynamic oral environment, emphasizing the importance of carefully assessing the correlation between laboratory findings and clinical applicability [47–49]. Future investigations should further explore the impact of aPDT on the adhesion performance of GFPs within root canals, irrespective of the PS and light source employed. Such studies are essential for elucidating the potential long-term benefits of aPDT in endodontic therapy, including the preservation of dental structures and oral rehabilitation.

## Conclusion

Based on the methods and findings of this laboratory study, the following conclusions can be drawn:

1. Antimicrobial photodynamic therapy with different photosensitizers, with or without calcium hydroxide, affected the push-out bond strength of glass-fiber posts to intraradicular dentin and the chemical composition of the root substrate;
2. The depth within the root canal influenced both the push-out bond strength of glass-fiber posts and the chemical composition of root dentin;
3. Curcumin at 500 mg/L is an effective photosensitizer for antimicrobial photodynamic therapy under blue LED light (λ = 480 nm), yielding favorable outcomes for push-out bond strength and glass-fiber posts sealing to intraradicular dentin, while maintaining consistent chemical composition across root dentin depths.

## References

[1] Gholami L, Shahabi S, Jazaeri M, Hadilou M, Fekrazad R (2023) Clinical applications of antimicrobial photodynamic therapy in dentistry. Front Microbiol 13:1020995. 10.3389/fmicb.2022.1020995 36687594 10.3389/fmicb.2022.1020995 PMC9850114

[2] Asnaashari M, Veshveshadi O, Aslani F, Hakimiha N (2023) Evaluation the antibacterial efficacy of sodium hypochlorite in combination with two different photodynamic therapy protocols against Enterococcus Faecalis in infected root canals: an in-vitro experiment. Photodiagnosis Photodyn Ther 43:103722. 10.1016/j.pdpdt.2023.103722 37487810 10.1016/j.pdpdt.2023.103722

[3] Barbosa-Ribeiro M, Arruda-Vasconcelos R, Louzada LM, Dos Santos DG, Andreote FD, Gomes BPFA (2021) Microbiological analysis of endodontically treated teeth with apical periodontitis before and after endodontic retreatment. Clin Oral Investig 25(4):2017–2027. 10.1007/s00784-020-03510-2 32860137 10.1007/s00784-020-03510-2

[4] Papalexopoulos D, Samartzi TK, Sarafianou A (2021) A thorough analysis of the Endocrown Restoration: A literature review. J Contemp Dent Pract 22(4):422–426 34267013

[5] Strazzi-Sahyon HB, Banci HA, Melo FS, Soares LS, Spin-Neto R, Duarte MAH, Cintra LTA, Dos Santos PH, Sivieri-Araujo G (2023) In vitro study on how photodynamic therapy and calcium hydroxide medication influence adhesive interface properties of glass-fiber posts to intraradicular dentin. J Mech Behav Biomed Mater 141:105757. 10.1016/j.jmbbm.2023.105757 36924612 10.1016/j.jmbbm.2023.105757

[6] Someya T, Kinoshita H, Harada R, Kawada E, Takemoto S (2017) Effects of calcium hydroxide reagent on the bond strength of resin cements to root dentin and the retention force of FRC posts. Dent Mater J 36(5):630–637. 10.4012/dmj.2016-355 28566675 10.4012/dmj.2016-355

[7] Strazzi-Sahyon HB, Banci HA, Maltarollo TFH, Martinez CMT, Rocha EA, Figueiredo RB, Garcia DCS, Constantino CJL, Spin-Neto R, Duarte MAH, Cintra LTA, Dos Santos PH, Sivieri-Araujo G (2024) The impact of methylene blue photosensitizer, aPDT and a calcium hydroxide-based paste on the physicochemical and mechanical characteristics of root canal dentin and the bonding interface of fiberglass posts. J Photochem Photobiol B 253:112878. 10.1016/j.jphotobiol.2024.112878 38447252 10.1016/j.jphotobiol.2024.112878

[8] Asnaashari M, Ashraf H, Rahmati A, Amini N (2017) A comparison between effect of photodynamic therapy by LED and calcium hydroxide therapy for root canal disinfection against Enterococcus faecalis: a randomized controlled trial. Photodiagnosis Photodyn Ther 17:226–232. 10.1016/j.pdpdt.2016.12.009 28040502 10.1016/j.pdpdt.2016.12.009

[9] Mahmoudi H, Bahador A, Pourhajibagher M, Alikhani MY (2018 Summer) Antimicrobial photodynamic therapy: an Effective Alternative Approach to control bacterial infections. J Lasers Med Sci 9(3):154–160. 10.15171/jlms.2018.29 30809325 10.15171/jlms.2018.29 PMC6378356

[10] El-Gendy AO, Ezzat S, Samad FA, Dabbous OA, Dahm J, Hamblin MR, Mohamed T (2024) Studying the viability and growth kinetics of Vancomycin-resistant Enterococcus faecalis V583 following femtosecond laser irradiation (420–465 nm). Lasers Med Sci 39(1):144. 10.1007/s10103-024-04080-5 38809462 10.1007/s10103-024-04080-5 PMC11136855

[11] Kandil H, Ahmed E, Fouad N, Ali Dabbous O, Niazy M, Mohamed T (2023) Using Femtosecond Laser Light-activated materials: the biomimetic dentin remineralization was monitored by Laser-Induced Breakdown Spectroscopy. Med (Kaunas) 59(3):591. 10.3390/medicina59030591 10.3390/medicina59030591 PMC10054410 36984592

[12] Ahmed E, El-Gendy AO, Hamblin MR, Mohamed T (2021) The effect of femtosecond laser irradiation on the growth kinetics of Staphylococcus aureus: an in vitro study. J Photochem Photobiol B 221:112240. 10.1016/j.jphotobiol.2021.112240 34130092 10.1016/j.jphotobiol.2021.112240

[13] Ahmed E, El-Gendy AO, Moniem Radi NA, Mohamed T (2021) The bactericidal efficacy of femtosecond laser-based therapy on the most common infectious bacterial pathogens in chronic wounds: an in vitro study. Lasers Med Sci 36(3):641–647. 10.1007/s10103-020-03104-0 32725427 10.1007/s10103-020-03104-0

[14] Alkahtany MF (2023) Efficacy of curcumin-mediated photodynamic therapy for root canal therapy procedures: a systematic review. Photodiagnosis Photodyn Ther 41:103252. 10.1016/j.pdpdt.2022.103252 36563708 10.1016/j.pdpdt.2022.103252

[15] Sivieri-Araujo G, Queiroz ÍOA, Fabbro RD, Esteves F, Cintra LTA, Duarte PCT, Bagnato VS, Oliveira SHP, Gomes-Filho JE (2017) Rat tissue reaction and cytokine production induced by antimicrobial photodynamic therapy. Photodiagnosis Photodyn Ther 18:315–318. 10.1016/j.pdpdt.2017.04.002 28389372 10.1016/j.pdpdt.2017.04.002

[16] Alves-Silva EG, Arruda-Vasconcelos R, Louzada LM, de-Jesus-Soares A, Ferraz CCR, Almeida JFA, Marciano MA, Steiner-Oliveira C, Bello-Silva MS, Shemesh H, Gomes BPFA (2022) The effect of photodynamic therapy on postoperative pain in teeth with primary endodontic infection. Photodiagnosis Photodyn Ther 37:102700. 10.1016/j.pdpdt.2021.102700 34954090 10.1016/j.pdpdt.2021.102700

[17] Alves-Silva EG, Arruda-Vasconcelos R, Louzada LM, de-Jesus-Soares A, Ferraz CCR, Almeida JFA, Marciano MA, Steiner-Oliveira C, Santos JMM, Gomes BP (2023) Effect of antimicrobial photodynamic therapy on the reduction of bacteria and virulence factors in teeth with primary endodontic infection. Photodiagnosis Photodyn Ther 41:103292. 10.1016/j.pdpdt.2023.103292 36681260 10.1016/j.pdpdt.2023.103292

[18] Nagata JY, Hioka N, Kimura E, Batistela VR, Terada RS, Graciano AX, Baesso ML, Hayacibara MF (2012) Antibacterial photodynamic therapy for dental caries: evaluation of the photosensitizers used and light source properties. Photodiagnosis Photodyn Ther 9(2):122–131. 10.1016/j.pdpdt.2011.11.006 22594982 10.1016/j.pdpdt.2011.11.006

[19] Strazzi-Sahyon HB, Cintra LTA, Nakao JM, Takamiya AS, Queiroz IOA, Dos Santos PH, Oliveira SHP, Sivieri-Araujo G (2022) Cytotoxicity of root canal irrigating solutions and photodynamic therapy using curcumin photosensitizer. Photodiagnosis Photodyn Ther 38:102795. 10.1016/j.pdpdt.2022.102795 35263668 10.1016/j.pdpdt.2022.102795

[20] Al Ahdal K, Al Deeb L, Al-Hamdan RS, Bin-Shuwaish MS, Al Deeb M, Maawadh AM, AlHelal A, Vohra F, Abduljabbar T (2020) Influence of different photosensitizers on push-out bond strength of fiber post to radicular dentin. Photodiagnosis Photodyn Ther 31:101805. 10.1016/j.pdpdt.2020.101805 32404299 10.1016/j.pdpdt.2020.101805

[21] Strazzi Sahyon HB, Silva PPD, Oliveira MS, Cintra LTA, Dezan-Júnior E, Gomes-Filho JE, Jacinto RC, Dos Santos PH, Sivieri-Araujo G (2019) Influence of curcumin photosensitizer in photodynamic therapy on the mechanical properties and push-out bond strength of glass-fiber posts to intraradicular dentin. Photodiagnosis Photodyn Ther 25:376–381. 10.1016/j.pdpdt.2019.01.025 30685549 10.1016/j.pdpdt.2019.01.025

[22] Almadi KH, Alkahtany MF, Almutairi B Influence of synthetic and natural photosensitizers activated by photodynamic therapy on extrusion bond strength of fiber post to radicular dentin. Pak J Med Sci 2021 Nov-Dec ;37(7):1912–1917. 10.12669/pjms.37.7.4331 10.12669/pjms.37.7.4331 PMC8613034 34912417

[23] Moradi M, Fazlyab M, Pourhajibagher M, Chiniforush N Antimicrobial action of photodynamic therapy on Enterococcus faecalis biofilm using curing light, curcumin and riboflavin. Aust Endod J 2022 Ago ;48(2):274–282. 10.1111/aej.12565 10.1111/aej.12565 34529329

[24] Strazzi-Sahyon HB, da Silva PP, Nakao JM, da Silva PZ, Nunes LP, Seron MA, Cintra LTA, Dos Santos PH, Sivieri-Araujo G (2021) Influence of two photodynamic therapy sessions and different photosensitizers on the bond strength of glass-fiber posts in different regions of intraradicular dentin. Photodiagnosis Photodyn Ther 33:102193. 10.1016/j.pdpdt.2021.102193 33503518 10.1016/j.pdpdt.2021.102193

[25] Yamamoto LY, Loureiro C, Cintra LTA, Leonardo RT, Banci HA, Ribeiro APF, Sivieri-Araujo G, Jacinto RC (2021) Antibiofilm activity of laser ablation with indocyanine green activated by different power laser parameters compared with photodynamic therapy on root canals infected with Enterococcus faecalis. Photodiagnosis Photodyn Ther 35:102377. 10.1016/j.pdpdt.2021.102377 34087469 10.1016/j.pdpdt.2021.102377

[26] Maawadh AM, Al Ahdal K, Al Deeb L, Alshamrani AS, Almohareb T, Alrahlah A (2023) Effectiveness of final cavity disinfectant terminalia chebula, malachite, and indocyanine green, against E. Faecalis and on the bond interface of fiber post to radicular dentin. Photodiagnosis Photodyn Ther 42:103538. 10.1016/j.pdpdt.2023.103538 37001714 10.1016/j.pdpdt.2023.103538

[27] Sivieri-Araujo G, Strazzi-Sahyon HB, Jacomassi DP, Dos Santos PH, Cintra LTA, Kurachi C, Bagnato VS (2022) Effects of methylene blue and curcumin photosensitizers on the color stability of endodontically treated intraradicular dentin. Photodiagnosis Photodyn Ther 37:102650. 10.1016/j.pdpdt.2021.102650 34838697 10.1016/j.pdpdt.2021.102650

[28] Strazzi Sahyon HB, Pereira da Silva P, Silva de Oliveira M, Angelo Cintra LT, Gomes-Filho JE, Henrique Dos Santos P, Sivieri-Araujo G (2018) Effect of photodynamic therapy on the mechanical properties and bond strength of glass-fiber posts to endodontically treated intraradicular dentin. J Prosthet Dent 120(2):317. 10.1016/j.prosdent.2018.05.009 10.1016/j.prosdent.2018.05.009 30097263

[29] Banci HA, Strazzi-Sahyon HB, Duarte M, Cintra L, Gomes-Filho JE, Chalub LO, Berton SA, de Oliveira V, Dos Santos PH, Sivieri-Araujo G (2020) Influence of photodynamic therapy on bond strength and adhesive interface morphology of MTA based root canal sealer to different thirds of intraradicular dentin. Photodiagnosis Photodyn Ther 32:102031. 10.1016/j.pdpdt.2020.102031 33011397 10.1016/j.pdpdt.2020.102031

[30] Allison RR, Moghissi K (2013) Photodynamic therapy (PDT): PDT mechanisms. Clin Endosc 46(1):24–29. 10.5946/ce.2013.46.1.24 23422955 10.5946/ce.2013.46.1.24 PMC3572346

[31] Strazzi-Sahyon HB, de Oliveira MS, da Silva PP, Banci HA, de Melo FS, Martinez CMT, Cintra LTA, Gomes-Filho JE, Dezan-Júnior E, Dos Santos PH, Sivieri-Araujo G (2020) Does photodynamic therapy with methylene blue affect the mechanical properties and bond strength of glass-fiber posts in different thirds of intraradicular dentin? Photodiagnosis Photodyn Ther 30:101673. 10.1016/j.pdpdt.2020.101673 31988021 10.1016/j.pdpdt.2020.101673

[32] Küçükkaya Eren S, Uzunoğlu E, Sezer B, Yılmaz Z, Boyacı İH (2018) Mineral content analysis of root canal dentin using laser-induced breakdown spectroscopy. Restor Dent Endod 43(1):e11. 10.5395/rde.2018.43.e11 29487841 10.5395/rde.2018.43.e11 PMC5816988

[33] Wang Z, Maezono H, Shen Y, Haapasalo M (2016) Evaluation of root canal dentin erosion after different irrigation methods using energy-dispersive X-ray spectroscopy. J Endod 42(12):1834–1839. 10.1016/j.joen.2016.07.024 27769680 10.1016/j.joen.2016.07.024

[34] Piai GG, Duarte MAH, Nascimento ALD, Rosa RAD, Só MVR, Vivan RR (2018) Penetrability of a new endodontic sealer: a confocal laser scanning microscopy evaluation. Microsc Res Tech 81(11):1246–1249. 10.1002/jemt.23129 30295382 10.1002/jemt.23129

[35] Moda MD, Fagundes TC, Briso ALF, Dos Santos PH (2018) Analysis of the bond interface between self-adhesive resin cement to eroded dentin in vitro. PLoS ONE 13(11):e0208024 30475892 10.1371/journal.pone.0208024 PMC6258132

[36] Alrefeai MH, Aljamhan AS, Alhabdan A, Alzehiri MH, Naseem M, Alkhudhairy F (2022) Influence of methylene blue, Riboflavin, and indocyanine green on the bond strength of caries affected dentin when bonded to resin-modified glass ionomer cement. Photodiagnosis Photodyn Ther 38:102792. 10.1016/j.pdpdt.2022.102792 35257973 10.1016/j.pdpdt.2022.102792

[37] Mirhashemi A, Janani R, Bahrami R, Chiniforush N (2022) Evaluation of the photodynamic therapy with riboflavin and curcumin on shear bond strength of orthodontic bracket: an in vitro study. Photodiagnosis Photodyn Ther 38:102787. 10.1016/j.pdpdt.2022.102787 35231617 10.1016/j.pdpdt.2022.102787

[38] Ballal NV, Mala K, Bhat KS (2011) Evaluation of decalcifying effect of maleic acid and EDTA on root canal dentin using energy dispersive spectrometer. Oral Surg Oral Med Oral Pathol Oral Radiol Endod 112(2):e78–84. 10.1016/j.tripleo.2011.01.034 21530335 10.1016/j.tripleo.2011.01.034

[39] Al-Kheraif AA, Mohamed BA, Khan AA, Al-Shehri AM (2022) Role of riboflavin; curcumin photosensitizers and ozone when used as canal disinfectant on push-out bond strength of glass fiber post to radicular dentin. Photodiagnosis Photodyn Ther 37:102592. 10.1016/j.pdpdt.2021.102592 34673270 10.1016/j.pdpdt.2021.102592

[40] Fernandes KGC, Silva BBD, Boer NC, Mandarini DR, Moreti LCT, Kato AS, Bueno CEDS, Limoeiro AGDS, Pinheiro SL, Martin AS, Fontana CE (2020) The effectiveness of three irrigation systems in the Enterococcus Faecalis reduction after instrumentation with a reciprocating instrument. Eur J Dent 14(4):539–543. 10.1055/s-0040-1714760 32898871 10.1055/s-0040-1714760 PMC7535969

[41] Mohammadi Z, Dummer PM (2011) Properties and applications of calcium hydroxide in endodontics and dental traumatology. Int Endod J 44(8):697–730. 10.1111/j.1365-2591.2011.01886.x 21535021 10.1111/j.1365-2591.2011.01886.x

[42] Zamparini F, Prati C, Taddei P, Spinelli A, Di Foggia M, Gandolfi MG (2022) Chemical-Physical properties and Bioactivity of New Premixed Calcium Silicate-Bioceramic Root Canal Sealers. Int J Mol Sci 23(22):13914. 10.3390/ijms232213914 36430393 10.3390/ijms232213914 PMC9692705

[43] Souza CC, Bueno CE, Kato AS, Limoeiro AG, Fontana CE, Pelegrine RA (2019 Mar-Apr) Efficacy of passive ultrasonic irrigation, continuous ultrasonic irrigation versus irrigation with reciprocating activation device in penetration into main and simulated lateral canals. J Conserv Dent 22(2):155–159. 10.4103/JCD.JCD_387_18 31142985 10.4103/JCD.JCD_387_18 PMC6519188

[44] de Oliveira RL, Guerisoli DMZ, Duque JA, Alcalde MP, Onoda HK, Domingues FHF, Vivan RR, Duarte MAH (2019) Computed microtomography evaluation of calcium hydroxide-based root canal dressing removal from oval root canals by different methods of irrigation. Microsc Res Tech 82(3):232–237. 10.1002/jemt.23164 30614119 10.1002/jemt.23164

[45] Singh S, Nagpal R, Manuja N, Tyagi SP (2015) Photodynamic therapy: an adjunct to conventional root canal disinfection strategies. Aust Endod J 41(2):54–71. 10.1111/aej.12088 25404404 10.1111/aej.12088

[46] Abu Zeid S, Edrees HY, Mokeem Saleh AA, Alothmani OS (2021) Physicochemical properties of two generations of MTA-Based Root Canal Sealers. Mater (Basel) 14(20):5911. 10.3390/ma14205911 10.3390/ma14205911 PMC8538924 34683503

[47] Strazzi-Sahyon HB, Oliveira AKL, Carvalho AP, Figueiredo RB, Cintra LTA, Gomes-Filho JE, Dos Santos PH, Sivieri-Araujo G (2021) Influence of photodynamic therapy and intracanal medication on Martens hardness, elastic modulus and bond strength of glass-fiber posts to endodontically treated root dentin. Photodiagnosis Photodyn Ther 36:102571. 10.1016/j.pdpdt.2021.102571 34626826 10.1016/j.pdpdt.2021.102571

[48] Almadi KH (2023) Impact of antimicrobial photodynamic therapy on the bond-strength and penetration of endodontic sealers: a systematic review. Photodiagnosis Photodyn Ther 41:103249. 10.1016/j.pdpdt.2022.103249 36563709 10.1016/j.pdpdt.2022.103249

[49] Capar ID, Aydinbelge HA (2013) Surface change of root canal dentin after the use of irrigation activation protocols: electron microscopy and an energy-dispersive X-ray microanalysis. Microsc Res Tech 76(9):893–896. 10.1002/jemt.22244 23760927 10.1002/jemt.22244

